# Absorption and Intestinal Metabolic Profile of Oleocanthal in Rats

**DOI:** 10.3390/pharmaceutics12020134

**Published:** 2020-02-05

**Authors:** Anallely López-Yerena, Anna Vallverdú-Queralt, Raf Mols, Patrick Augustijns, Rosa M. Lamuela-Raventós, Elvira Escribano-Ferrer

**Affiliations:** 1 Nutrition, Food Science and Gastronomy Department, XaRTA, Institute of Nutrition and Food Safety (INSA-UB), School of Pharmacy and Food Sciences, University of Barcelona, 08028 Barcelona, Spain; naye.yerena@gmail.com (A.L.-Y.); avallverdu@ub.edu (A.V.-Q.); lamuela@ub.edu (R.M.L.-R.); 2 CIBER Physiopathology of Obesity and Nutrition (CIBEROBN), Institute of Health Carlos III, 28029 Madrid, Spain; 3 Drug Delivery and Disposition, KU Leuven, 3000 Leuven, Belgium; raf.mols@kuleuven.be (R.M.); patrick.augustijns@pharm.kuleuven.be (P.A.); 4 Department of Pharmacy and Pharmaceutical Technology and Physical Chemistry, Biopharmaceutics and Pharmacokinetics Unit, Institute of Nanoscience and Nanotechnology (IN2UB), Pharmacy and Food Sciences School, University of Barcelona, 08028 Barcelona, Spain

**Keywords:** bioavailability, metabolism, in situ perfusion, permeability, extra virgin olive oil, secoiridoids

## Abstract

Oleocanthal (OLC), a phenolic compound of extra virgin olive oil (EVOO), has emerged as a potential therapeutic agent against a variety of diseases due to its anti-inflammatory activity. The aim of the present study is to explore its in vivo intestinal absorption and metabolism. An in situ perfusion technique in rats was used, involving simultaneous sampling from the luminal perfusate and mesenteric blood. Samples were analysed by UHPLC–MS–MS for the presence of oleocanthal (OLC) and its metabolites. OLC was mostly metabolized by phase I metabolism, undergoing hydration, hydrogenation and hydroxylation. Phase II reactions (glucuronidation of hydrogenated OLC and hydrated metabolites) were observed in plasma samples. OLC was poorly absorbed in the intestine, as indicated by the low effective permeability coefficient (2.23 ± 3.16 × 10^−5^ cm/s) and apparent permeability coefficient (4.12 ± 2.33 × 10^−6^ cm/s) obtained relative to the values of the highly permeable reference compound levofloxacin (LEV). The extent of OLC absorption reflected by the area under the mesenteric blood-time curve normalized by the inlet concentration (AUC) was also lower than that of LEV (0.25 ± 0.04 vs. 0.64 ± 0.03, respectively). These results, together with the observed intestinal metabolism, suggest that OLC has a moderate-to-low oral absorption; but higher levels of OLC are expected to reach human plasma vs. rat plasma.

## 1. Introduction

The secoiridoids are the most abundant and complex family of phenolic compounds in extra virgin olive oil (EVOO). The main secoiridoids compounds identified in EVOO are the monoaldehydic forms of oleuropein (3,4-DHPEA-EA) and ligstroside aglycones (*p*-HPEA-EA) and the dialdehydic forms of their decarboxymethylated derivatives, oleacein (3,4-DHPEA-EDA) and oleocanthal (*p*-HPEA-EDA) [1]. Since its identification in 1993 [2], oleocanthal (OLC) has been targeted by numerous in vitro and in vivo studies aiming to understand the health effects of EVOO consumption [3]. The data obtained on OLC thus far have clearly demonstrated its anti-inflammatory activity [4] as well as its role in the prevention of various pathologies with an inflammatory component [5]. OLC also exerts a neuroprotective effect in conditions such as Alzheimer’s disease [6,7] and has shown promising chemotherapeutic properties, reducing cell proliferation and promoting cell death through different mechanisms of action [8]. Antirheumatic activity has been demonstrated in in vitro studies, in which OLC ameliorated osteoarthritis and rheumatoid arthritis [3]. Additionally, OLC has been shown to be beneficial for cardiovascular health, improving endothelial function in patients with early atherosclerosis and reducing platelets in healthy men [3].

Given this broad and promising range of biological effects, most research on OLC has been focused on its health properties and the agronomic and processing factors that promote its presence in EVOO [9]. Information on the bioavailability of this phenolic compound would help determine the mechanisms behind its biological activities; however, none have been reported to date. The bioavailability of ingested phenolic compounds can be influenced by factors such as diet, genomic profile, enzymatic activity and colonic microflora [3], but it also depends on the extent of absorption and metabolism after ingestion. In this context, many in vitro and in vivo models for estimating human intestinal permeability and first-pass metabolism have been developed [10,11].

Before reaching the bloodstream, drugs and nutrients administered orally are usually absorbed from the small intestine. Although different factors (physicochemical, physiological and the matrix effect) can affect the rate and extent of absorption, the absorption of orally administered drugs is mainly determined by the solubility/dissolution of the molecule in the gastrointestinal environment as well as the permeability of the gastrointestinal wall [12]. In situ intestinal perfusion with venous sampling models facilitates a direct determination of drug absorption through the enterocytes on the basis of appearance kinetics in pre-hepatic blood [13]. This experimental model, which includes a mucus layer, blood irrigation and innervation, has been widely used due to its similarity with in vivo conditions [14]. It also allows the study of the role of transporters in absorption through the biorelevant expression of proteins (both transporters and intestinal enzymes) [12,15] and has full metabolic capacity for up to 120 min after intestinal isolation [16].

The objectives of the present work are to study the intestinal absorption and metabolism of OLC using an in situ perfusion technique in rats involving simultaneous sampling from the luminal perfusate and mesenteric blood. As a reference, the highly permeable drug levofloxacin (LEV) was included in the study. In addition, an intestinal metabolic profile of OLC was determined.

## 2. Materials and Methods

### 2.1. Reagents and Materials

OLC was purchased from PhytoLab GmbH (Vestenbergsgreuth, Germany). Phenol red, LEV, heparin sodium salt from porcine intestinal mucosa, Hanks’ Balanced Salt Solution (HBBS) and HEPES 1 M solution were obtained from Sigma Aldrich (Madrid, Spain). Pentobarbital sodium 200 mg/mL (Dolethal) was purchased from Vétoquinol (Madrid, Spain) and isoflurane from Laboratorios Esteve (Barcelona, Spain). The reagents, methanol, acetonitrile and formic acid, were purchased from Sigma-Aldrich.

### 2.2. Animals

These studies were conducted following a protocol approved by the Animal Experimentation Ethics Committee of the University of Barcelona, Spain (trial no. CEEA 124/16) and Generalitat de Catalunya (no. 6435, 27 June 2019). For each compound, four Sprague–Dawley rats (body weight 306 ± 31 g) were used per intestinal perfusion with the mesenteric blood sampling experiment, and four (~350–400 g) were used as blood donors.

### 2.3. Single-Pass Intestinal Perfusion Studies

OLC was assayed at 0.1 mg/mL (328.94 µM) in transport medium (TM pH 7, 9.7 g/L HBSS buffered with HEPES 10 mM). This concentration was chosen by taking into account both the OLC concentration in EVOO (around of 200 mg/kg) and the daily ingestion of EVOO recommended by the European Food Safety Authority (EFSA) (at least 5 mg of hydroxytyrosol and its derivatives per 20 g of olive oil) [17] and also considering the limit of quantification of the analytical technique. LEV was assayed at 3 mg/mL, according to its high dose strength, in 250 mL TM.

Single-pass intestinal perfusion was performed in anesthetized rats according to the method described by Brouwers et al. [15], with simultaneous sampling from the luminal perfusate and mesenteric blood.

#### 2.3.1. Donor Blood

On the day of the perfusion, one or two rats (350–400 g) were anesthetised by isoflurane inhalation, and the whole blood was collected via cardiac puncture. The blood was diluted with heparin (50 u/mL TM) to 80% blood and kept in a 20 mL syringe for the in situ intestinal perfusion experiment.

#### 2.3.2. Surgical Procedure

Anaesthesia was induced by intraperitoneal injection of pentobarbital sodium (60 mg/kg BW). The rats were then placed on a homeothermic blanket to maintain the body temperature at 37 °C. First, under general anaesthesia, the left jugular vein was cannulated with a heparinised (50 IU/mL) 0.5 × 0.9 mm/20 G catheter (Cavafix^®^ MT, Braun Medical S.A., Barcelona, Spain) for infusion of donor blood. Second, a midline abdominal incision was made to isolate approximately 10 cm of the ileum using the ileocecal junction as a point of reference. Two glass cannulas (o.d. 4 mm, i.d. 3 mm, Duran^®^, Vidrafoc, Barcelona, Spain) were inserted at the proximal and distal ends of the isolated segment. The segment was rinsed by injection TM to remove the content until the solution came out clear. Next, the inlet cannula was connected to polyethylene tubing (i.d. 3 mm), which aspirated the tested solution (OLC or LEV) using a peristaltic pump (Minipuls3, M312 model, Gilson, Le Bel, France). Finally, the mesenteric vein draining blood from the intestinal segment was cannulated by inserting approximately 1 cm of a catheter (BD Insyte-W 24GA 0.7 × 19 mm, Becton Dickinson, Sangüesa S.A., Cornellà de Llobregat, Spain). The cannula was secured with a knot and connected to a silicone tube (i.d. 0.64 mm; o.d. 1.19 mm, Freudenberg Medical Europe, VWR International Eurolab S.L., Llinars del Vallés, Spain) that allowed blood to flow into the heparinised tubes. Care was taken to handle the small intestine gently to maintain normal blood supply. The exposed areas (both the jugular and abdomen) were wet with TM at 37 °C and covered with Parafilm^®^ to keep them warm and moistened during the experiment.

#### 2.3.3. Intestinal Perfusion

The experiments were carried out in a laboratory room with infrared light to avoid oxidation of OLC. The procedure was started by delivering the perfusion solution containing OLC (0.1 mg/mL) or LEV (3 mg/mL) (in separate experiments) and phenol red (0.1 mg/mL) at a flow rate of 1 mL/min to the intestinal segment previously cannulated. Simultaneously, the blank donor rat blood was supplied at a rate of 0.3 mL/min using a syringe pump (Injectomat MC Agilia, Fresenius Kabi España, Barcelona, Spain). Samples of both the intestinal lumen and mesenteric blood were collected at the same time. Simultaneously, the outflow perfusate was collected in 1.5 mL amber vials at 5 min intervals for 60 min, and the blood was collected in pre-weighted lithium-heparinised tubes (BD Vacutainer). At the end of the experiment, the animal was euthanised by injection of air into the jugular, and the length of the intestinal segment was measured. Next, the perfusion samples were centrifuged (7516 g for 10 min at 4 °C), and the supernatant was stored at −80 °C until analysis. The tubes with blood samples were weighted and centrifuged (7516.3 g for 10 min at 4 °C), and the plasma was collected and stored at −80 °C until solid-phase extraction (SPE).

The stability of the test products, sampled at different times, was tested in the perfusion solution at 37 °C for 60 min.

### 2.4. Oleocanthal Analysis

#### 2.4.1. Solid-Phase Extraction (SPE) of OLC from Plasma

Extraction of OLC and its metabolites from plasma samples was carried out using an Oasis^®^ HLB 30-mm (30 mg) 96-well plate (Waters, Wexford, Ireland), following the methodology proposed by Orrego-Lagarón and colleagues [18], with some modifications. Initially, the samples were thawed and centrifuged (7516 g, 10 min at 4 °C). The pre-treatment of samples consisted of diluting an exact volume of the supernatants to 1 mL with 0.1% of formic acid in water (*v*/*v*). The plate was activated with methanol and water containing 1% (*v*/*v*) formic acid. After loading the plasma mixture, the plates were cleaned by adding 1 mL 1% formic acid in water, followed by 1 mL methanol/water (5:95). The retained compounds were then eluted with 1 mL methanol acidified with 0.1% formic acid (*v*/*v*), and the resulting fraction was evaporated to dryness at room temperature under a stream of N_2_. Finally, the samples were reconstituted with 100 µL of methanol containing 0.1% (*v*/*v*) formic acid. The extraction was performed in a darkened room with a red safety light to avoid oxidation of the analytes.

#### 2.4.2. LC–ESI–LTQ–Orbitrap–MS

An LTQ Orbitrap Velos mass spectrometer (Thermo Scientific, Hemel Hempstead, UK) equipped with an ESI source in negative mode was used for accurate mass measurements. The operation parameters were the following: source voltage, 4 kV; capillary temperature, 275 °C (FT Automatic gain control (AGC) target 5 × 10^5^ for MS mode and 5 × 10^4^ for MS^n^ mode). The arbitrary units were used for sheath gas, auxiliary gas and sweep gas (20, 10 and 2, respectively). All samples were analysed in full scan mode at a resolving power of 30,000 at *m*/*z* 400, and data-dependent MS/MS events were acquired at a resolving power of 15,000. The most intense ions detected during full scan MS triggered data-dependent scanning. Ions that were not intense enough for a data-dependent scan were analysed in MS^n^ mode with the same orbitrap resolution (15,000 at *m*/*z* 400). An isolation width of 100 amu was used, and precursors were fragmented by collision-induced dissociation C-trap (CID) with normalized collision energy (35 V) and an activation time of 10 ms. The mass range in FTMS mode was from *m/z* 100 to 1000. The system was controlled by XCalibur software v2.0.7 (ThermoFisher Scientific).

An Accela chromatograph (Thermo Scientific, Hemel Hempstead, UK) equipped with a quaternary pump, a photodiode array detector (PDA) and a thermostated autosampler was used for the liquid chromatography analysis. The injection volume was 2 µL, the flow rate was set to 0.6 mL/min and the separations were carried out on an Acquity^TM^ UPLC^®^ BEH C18 Pre-Column (2.1 × 5 mm, i.d., 1.7 µm particle size) and an Acquity^TM^ UPLC^®^ BEH C18 Column (2.1 × 50 mm, i.d., 1.7 µm particle size) (Waters Corporation^®^, Wexford, Ireland) at 50 °C. The mobile phases consisted of methanol (A) and H_2_O (B), both with 0.1% of formic acid. The elution was performed by means of an increasing linear gradient (*v*/*v*) of B (*t* (min), %B), as follows: (0, 100); (2, 100); (6, 46.4); (8, 0); (9, 0); (9.1, 100); (11, 100).

#### 2.4.3. UHPLC–ESI–MS/MS

All luminal, plasma and stability samples were analysed by ultra-high-performance liquid chromatography/electrospray ionization tandem mass spectrometry (UHPLC–ESI–MS/MS). The quantification of OLC and its metabolites was performed using an Acquity^TM^ UPLC (Waters; Milford, Massachusetts, USA) coupled to an API 3000 triple-quadruple mass spectrometer (ABSciex, Concord, Ontario, Canada) with a turbo ion spray source. The chromatographic separation of the parent compound and its metabolites were achieved using the same conditions (flow rate, column, injection volume and gradient conditions) as in LC–ESI–LTQ–Orbitrap–MS (Section 2.4.2).

The ionization of all compounds was performed using an electrospray interface operating in the negative mode [M–H] in the multiple monitoring mode (MRM). The arbitrary units were used for the nebulizer (10), curtain (12) and drying gas (450 °C) using N_2_; the capillary voltage was −3500 V. The declustering, focusing and entrance potential and the collision energy were optimised to detect OLC and oleacein with the highest signal (see Table S1 for a detailed description of each compound). The system was controlled by Analyst version 1.4.2 software supplied by Applied Biosystems.

The calibration curves were prepared in TM and rat plasma (0–150 and 0–50 µg/mL, respectively) using OLC. The metabolites—in the absence of the reference standard—were evaluated by a screening method that includes a relative comparison of metabolite abundance (peak area ratio of metabolite/dosed parent compound OLC) [19,20].

### 2.5. Levofloxacin Analysis

A validated HPLC method was used to determine the concentration of LEV in luminal and plasma samples. The HPLC system consisted of two Waters 515 pumps, a 717 plus Waters auto sampler and a Waters dual absorbance detector 2487 with UV detection (Waters Chromatography S.A., Barcelona, Spain). The separation was carried out using a Hypersil^®^ Elite C18 column (150 mm × 4.6 mm i.d., particle size 5 µm, ThermoFisher Scientific) with the flow rate at 0.8 mL/min. The mobile phase was isocratic, consisting of 1% triethylamine (pH 3.0 adjusted with phosphoric acid) and acetonitrile (86/14, *v*/*v*). The UV detection wavelength was set at 293 nm. The run time was 10 min for lumen samples and 12 min for plasma samples, and the injection volume was 10 µL. The calibration curves were prepared in TM and plasma and were linear over the concentration ranges 5–100 and 10–100 µg/mL, respectively.

For plasma samples, the procedure of Watabe et al. with some modifications was followed [21]. The standard solutions for the calibration curves were prepared from three stock standard solutions, using LEV (200 µg/mL and 1 mg/mL in TM) and ciprofloxacin as the internal standard (1 mg/mL in HCL 0.1 N). Different volumes of the LEV stock solutions (between 20 and 80 µL) and 16 µL of ciprofloxacin (1 mg/mL) were added to Eppendorf tubes, and drug-free rat plasma was incorporated to a total volume of 200 µL.

A liquid–liquid extraction was achieved using 200 µL calibration standards or samples mixed with 100 µL 6% (*w*/*v*) perchloric acid. Each mixture was vortexed for 30 s. Then, 100 µL of methanol was added to the above mixture, which was vortexed again for 30 s. The mixture was centrifuged at 9900 rpm for 12 min at room temperature, and 10 µL of the supernatant was injected into the HPLC. The final concentration of ciprofloxacin in the samples was 40 µg/mL. The retention times for LEV and ciprofloxacin were 7.8 ± 0.2 min and 8.7 ± 0.3 min, respectively.

### 2.6. Phenol Red Analysis

The concentration of phenol red in luminal samples from the in situ experiments with LEV was determined using a colorimetric assay. In each microplate well, 200 µL samples diluted 1/10 were added. The dilution was prepared with a 50 µL sample, NaOH 1 N (20 µL) and TM (430 µL). The absorbance at 558 nm was measured with a multidetector microplate reader (Biotek Instruments Inc., Winooski, VT, USA) equipped with Gen5 2.00 data analysis software. The calibration curves of phenol red were prepared in the same dissolution medium as the luminal samples at the concentrations of 24, 12, 6 and 3 µg/mL.

The concentration of phenol red in lumen samples from the in situ experiments with OLC was analysed by UHPLC–ESI–MS/MS in the same conditions as the OLC.

### 2.7. Data Analysis

The effective permeability coefficient (*P*_eff_, cm/s) through the rat ileum was determined by Equation (1):

(1) $$P_{\mathrm{eff}}=\frac{-\emptyset_{\mathrm{in}}}{2\pi RL}\times Ln\;\frac{C_{\mathrm{in}}}{C_{\mathrm{out}.\mathrm{cor}}}$$

where $\emptyset_{\mathrm{in}}$ is the perfusion solution flow (1 mL/min), *C*_in_ and *C*_out.cor_ are the respective inlet and corrected outlet steady-state concentrations of the tested product, *R* is the radius of the intestinal segment (set to 0.2 cm) and *L* is the length of the intestinal segment determined after completion of the perfusion experiment.

The outlet concentrations were corrected for water transport by measuring the nonabsorbed and nonmetabolized phenol red marker according to Equation (2):

(2) $$C_{\mathrm{out}.\mathrm{cor}}=C_{\mathrm{out}}\times \frac{CPR_{\mathrm{in}}}{CPR_{\mathrm{out}}}$$

where *C*_out_ is the concentration of OLC or LEV in the perfusate at the specified time interval and *CPR*_in_ and *CPR*_out_ is the phenol red concentration in the inlet and outlet buffer solution at the specific time interval, respectively.

The apparent permeability coefficient (*P*_app_, cm/s) was calculated using Equation (3):

(3) $$P_{\mathrm{app}}=\frac{dQ}{dt}\times \frac{1}{A\times C_{0}}$$

where *Q* is the cumulative amount of OLC or LEV appearing in the mesenteric blood as a function of time *t* in steady-state conditions, *A* is the surface area of the exposed intestinal segment and *C*_0_ is the OLC or LEV initial concentration in the perfusate.

All in situ perfusion experiments were replicated in four rats, and the results were normalised to a 10-cm intestinal segment.

As a measure of the extent of absorption, the areas under the plasma concentration (normalised by the inlet concentration) time curves (AUC) for the intestinal perfusions were calculated using WinNonlin Professional software version 3.3 (Pharsight Corporation, Mountain View, USA) from time 0 to 60 min using the linear trapezoidal method.

Data are presented as mean and standard deviation (SD). Statistical analysis was performed using SAS (version 9.4). Statistical differences in the peak area ratio metabolite/parent OLC between different time periods were analysed using a one-way ANOVA. The *P*_eff_, *P*_app_ and AUC of OLC and LEV were compared using a Mann–Whitney *U*-test. Differences were considered significant at *p* < 0.05.

## 3. Results and Discussion

### 3.1. Intestinal Metabolic Profile of Oleocanthal

#### 3.1.1. Identification of Metabolites

**Figure 1:**
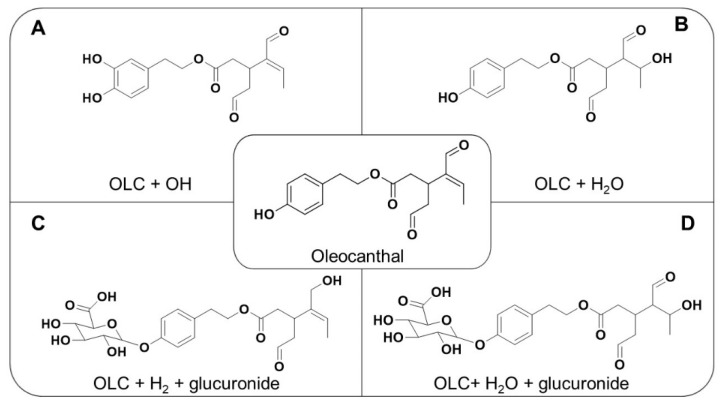
Chemical structure of oleocanthal and metabolites (**A**) = hydroxylated OLC; (**B**) = hydrated OLC; (**C**) = hydrogenated glucuronide; (**D**) = hydrated glucuronide).

To identify the OLC metabolites in plasma and perfusion samples, we initially used LC–ESI–LTQ–Orbitrap–MS in FTMS mode, followed by a data-dependent scan. Combining the FTMS scan and MSn experiments, four metabolites were identified in plasma and two metabolites (hydroxylated and hydrated) in perfusion samples. The proposed chemical structures are depicted in Figure 1. These metabolites and their retention times (RT), accurate masses, major fragments, error (mDa) and molecular formulas are shown in Table 1. Metabolite identification is important to reveal possible metabolic instability, causing extensive first-pass intestinal metabolism and poor oral bioavailability [22]. 

#### 3.1.2. Phase I Metabolism

**Figure 2:**
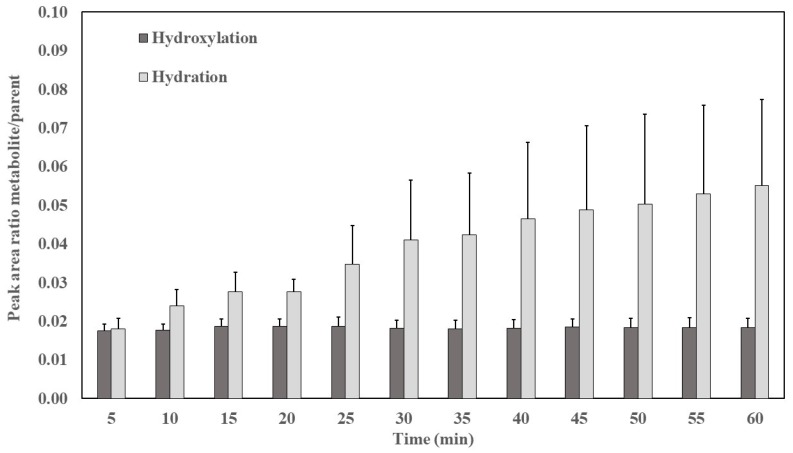
Peak area ratio metabolite/oleocanthal (OLC) as a function of time in lumen. Results are expressed as the mean ± standard deviation.

**Figure 3:**
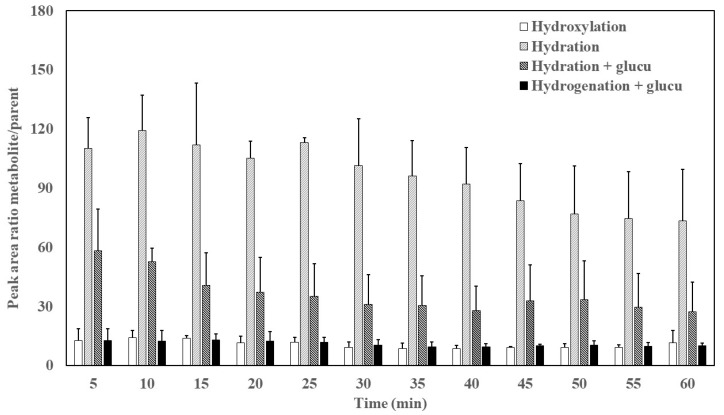
Peak area ratio metabolite/OLC as a function of time in plasma. Results are expressed as the mean ± standard deviation.

The intestine is the most important extrahepatic site for drug biotransformation [10]. When crossing enterocytes, EVOO phenolic compounds are subjected to extensive first-pass metabolism through phase I/II biotransformation [23]. In our study, the two metabolites corresponding to phase I metabolism (hydration and hydroxylation identified in perfusion and plasma samples) are shown in Figure 2 and Figure 3, respectively. Compared to OLC, the relative abundance of metabolites was higher in plasma than in the lumen.

These results are consistent with the work of Garcia-Villalba et al. [24], who studied the urinary excretion of phenolic compounds after olive oil intake in humans. They identified, among others, hydroxylated and hydrated metabolites from phase I reactions in urine; however, the samples were discarded because the metabolites could have been related to hydroxytyrosol derivatives. Hydrogenated and hydrated (+glucuronidation) metabolites were additionally identified from phase II reactions with the same accurate mass. In the current study, we can confirm that they were direct metabolites of OLC. In another study [25], the hydrated metabolite was also identified in plasma samples after the ingestion of EVOO.

As shown in Figure 2 and Figure 3, the most abundant phase I metabolite found in the samples was the hydrated form, with higher levels in plasma than in the lumen (*p* < 0.05). The metabolites in the lumen can originate from the microbiome, since many bacterial cytochromes P450 (CYP) are soluble [26], or from CYP of the enterocytes. This family of metabolic enzymes are present in the epithelium of the small intestine [27] and are responsible for the oxidative biotransformation of xenobiotics and other compounds [28]. The absorbed and metabolized OLC would be subsequently secreted to the intestinal lumen by efflux transporters.

The hydrogenated metabolites were found in a glucuronidated form in plasma but not in the lumen. Even when considering the differences between rat and human microsomal activity, hydrogenation seems to be an important phase I metabolic route for secoiridoids [25]. This reduction reaction is catalysed by NADPH-dependent aldo-keto reductases (AKR) located in the small intestine epithelium and can occur because OLC contains an open dialdehydic form of the attached elenolic acid molecule. During phase I metabolism, the reduction of aldehydes and ketones to primary and secondary alcohols, respectively, are formal functionalisation reactions and are involved in endogenous and xenobiotic compounds that have these carbonyl groups [29]. Moreover, the redox potential in the intestine favours the reduction reaction due to low oxygen tension, which provides a reducing environment, whereas oxidation is favoured in tissues such as the liver [30].

#### 3.1.3. Phase II Metabolism

The appearance in plasma of metabolites arising from phase II reactions (glucuronidation of hydrogenated and hydrated metabolites) from time 0 (at the start of the in situ intestinal perfusion) to 60 min are depicted in Figure 3. The main phase II metabolite detected in plasma samples was the hydrated metabolite with constant relative abundance over time (*p* < 0.05). 

**Figure 4:**
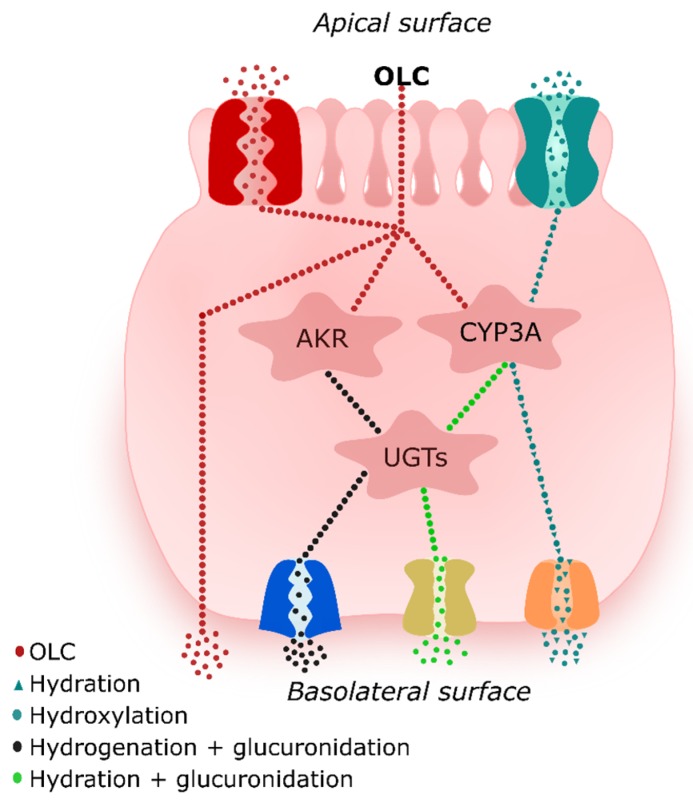
Tentative interactions of oleocanthal (OLC) with metabolic enzymes and transporters.

The glucuronidation of hydrogenated and hydrated metabolites likely occurs after oxidative-reductive metabolism because although drugs can undergo phase I and II reactions simultaneously, one of the pathways usually dominates [31]. The glucuronidation reaction mediates the transfer of a glucuronyl moiety from the ubiquitous co-substrate UDP-glucuronic acid to hydrophobic molecules with one or more electrophilic groups which serve as acceptors [32]. Similar results indicating that secoiridoids (3,4-DHPEA-EDA and 3,4-DHPEA-EA) undergo hydrogenation followed by glucuronidation have been previously reported in perfused segments of jejunum and ileum in rats [16]. Based on their high polarity and molecular weight, both phase II metabolites would be secreted to the mesenteric blood by transporters. A proposal of what could happen at the intestinal level is shown in Figure 4.

### 3.2. Perfusion Experiments and Intestinal Permeability of Oleocanthal

Prior to carrying out the intestinal permeability study, it is necessary to check the stability of the test compound and the reference standard in the perfusion solution at 37 °C during the test time (1 h). Both OLC and LEV remained stable in the perfusion solution (*p* < 0.05) during the experiments (Figure S1).

The intestinal permeability coefficient (*P*_eff_) is widely used as part of a general screening process for orally administered drugs to study their intestinal absorption. It is important to determine the intestinal permeability of OLC, a component of EVOO (and therefore of the Mediterranean diet), of which many beneficial health properties have been reported [33]. In this work, to assess both the intestinal absorption and potential intestinal first-pass metabolism of OLC, simultaneous samples were collected from both the intestinal lumen and the mesenteric circulation. The concentration of OLC and the relative abundance of metabolites was determined (see Section 3.1). In addition, to ascertain if the results obtained for OLC imply high or low absorption/permeability, a group of rats perfused with LEV was also included in the study. According to the current Biopharmaceutics Classification System (BCS) [34], LEV is a highly permeable drug with an oral bioavailability close to 100% [35,36]. When assayed at 3 mg/mL in Caco-2 cells [35], it produced an apparent permeability coefficient similar to that of metoprolol, another reference drug of high permeability.

Surprisingly, despite the myriad of potential health benefits attributed to EVOO phenolic compounds, there is currently little information available on the intestinal permeability coefficients of secoiridoids, especially OLC. Previous studies in rats have observed poor absorption of secoiridoids in the intestine perfused with oleuropein in iso-osmotic conditions, although it was significantly greater under hypotonic conditions (1.47 × 10^−6^ and 5.92 × 10^−6^ cm/s, respectively) [37]. Differentiated Caco-2 cell monolayers as a model system have been demonstrated to be highly permeable for hydroxytyrosol (12.4 ± 0.9 × 10^−6^ cm/s) [38].

The permeability coefficients of OLC (0.1 mg/mL) and LEV (3 mg/mL) in the rat ileum, taking into account the disappearance of the test product in the lumen (*P*_eff_) and appearance in the mesenteric circulation (*P*_app_), are shown in Table 2.

**Figure 5:**
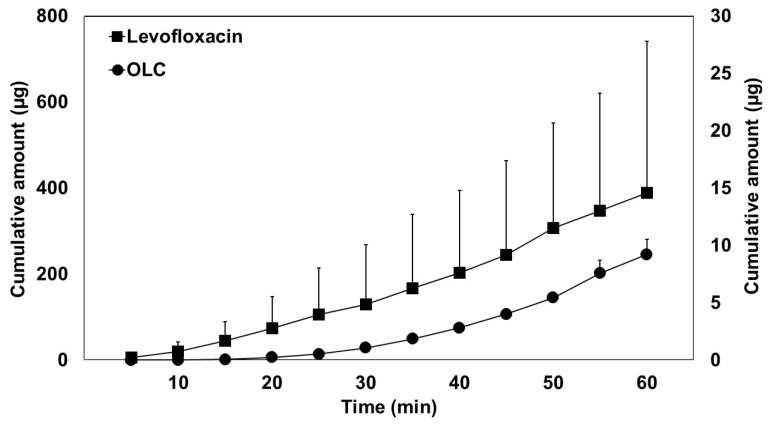
Transport of oleocanthal (OLC) (right *Y*-axis scale) and levofloxacin (left *Y*-axis scale) from the intestinal lumen to the mesenteric blood. Cumulative amount values are normalised to a 10-cm intestinal segment. Results are expressed as the mean ± standard deviation.

In Figure 5, the mean cumulative transport of OLC (A) and LEV (B), corrected for the length of the perfused segment, is illustrated as a function of time.

The difference between *P*_eff_ vs. *P*_app_ values is common in these types of studies; it has even been observed for theophylline where an absence of mass balance was observed, taking into account the mass lost from the perfusate and mass appearance in portal plasma [39]. Drug transfer from the lumen to the blood is not an instantaneous process: *P*_eff_ only refers to the resistance to transport from the lumen to the inside of the membrane, but not to the blood [40,41]. During the transport from the intestinal wall to the blood, different processes can occur, including accumulation in gut tissue, binding to transporters, gut wall metabolism, binding to red blood cells, and lymphatic transport [39]. Moreover, the rate of appearance in blood may be markedly dependent upon the blood flow rate. The difference in LEV values can be attributed to P-glycoprotein (P-gp) binding [42,43] and in the case of OLC, to microbiota and gut wall metabolism, according to the results reported in the previous section (Section 3.1). Moreover, as shown in Figure 5, the two compounds presented different accumulation profiles in mesenteric blood, with a lag time observed for OLC. This lag could be explained by the transit time in gut tissue due to phase I and phase II metabolic reactions in the enterocytes, which would delay appearance in the blood.

In our study, the decrease in concentration of LEV in the lumen samples was very low, resulting in lower absorption and permeability coefficient than expected. This low permeability could be attributed to LEV acting as a substrate for the efflux transporter P-gp, which would secrete the absorbed drug to the intestinal lumen. However, this finding observed in our in vivo study did not translate into a lower value of the apparent permeability coefficient in a study by Volpe [33] in in vitro Caco-2 cells, where a *P*_app_ (apical-basolateral direction) similar to the highly permeable standard metoprolol was obtained.

Nevertheless, in this study, the values of *P*_app_ obtained for OLC are lower (*p* < 0.05) than for LEV. The lower extent of absorption is also reflected in the AUC calculated from the plasma concentrations in mesenteric blood (normalized by the input concentration). The lower OLC AUC values (AUC 0.25 ± 0.04 vs. 0.635 ± 0.03 for OLC and LEV, respectively) confirm that the intestinal membrane had low permeability for OLC, although its physicochemical characteristics (log*P* = 1.15 and MW 304.34 g/mol) were not unfavourable for its absorption. The poor absorption of OLC in our experimental conditions (perfusion flow rate 1 mL/min), as well as its presence in the ileum (located next to the caecum, where bacterial density is high), could favour microbiome metabolism. Both circumstances, low absorption and high intestinal metabolism, can lead to incomplete bioavailability through a variety of mechanisms, including pre-systemic gastrointestinal and hepatic first-pass metabolism, saturation of carrier-mediated uptake processes, a low permeability constant, and P-gp-mediated transport out of the epithelial cells back into the lumen of the gastrointestinal tract [44]. According to its physicochemical properties (low solubility and relatively high lipophilicity), this compound meets the characteristics of BCS class 2. For these compounds, efflux transporters can affect their extent and rate of absorption [45], as observed in our study. The hypothesis that OLC could be a P-gp substrate should not be discarded, but more studies are needed to confirm it.

It should be noted that the human *P*_eff_ estimated for drugs transported by passive diffusion is, on average, 3.6 times higher in human in vivo than in rat in situ [11]; thus, higher levels of OLC are expected to reach human plasma.

## 4. Conclusions

In conclusion, OLC is a phenolic compound with an incomplete oral bioavailability due to its relatively low absorption (16%) and high intestinal metabolism. Its poor absorption was indicated by a low effective permeability coefficient, apparent permeability coefficient and area under the mesenteric blood–time curve normalized by the inlet concentration in comparison with the reference standard compound LEV. However, previous research has indicated that higher levels of OLC reach human plasma than in rats [11]. Regarding the metabolic profile, OLC was mostly metabolised by phase I hydration reactions. Hydrogenation and hydroxylation were also observed, and the hydrogenated and hydrated metabolites were then glucuronidated in phase II reactions.

This is the first in vivo study in rats to simultaneously assess the absorption and intestinal metabolism of OLC. Given the importance of phenolic compounds from EVOO, especially OLC on human health and its demonstrated moderated ability to be transported through the intestinal membrane, the development of dietary supplements containing OLC and excipients that favour their immediate release and absorption in the gastrointestinal tract should be considered, especially for populations with low intake of EVOO. In addition, further studies on OLC oral absorption and bioavailability are necessary, as well as studies on the biological relevance of the most abundant OLC metabolites.

## Figures and Tables

**Table 1:** Oleocanthal (OLC) and its metabolites identified in lumen and plasma samples using an LTQ–Orbitrap.

Compound	RT (min)	Accurate Mass	Major Fragments	Error (mDa)	Molecular Formula
OLC	5.8	303.1230	285.1125/179.0708	0.48	C_17_H_19_O_5_
OLC + OH	6.49	319.1180	153.0271/183.0665	0.16	C_17_H_19_O_6_
OLC + H_2_O	6.36	321.1344	201.0759/183.0665	0.88	C_17_H_21_O_6_
OLC + H_2_ + glucuronide	5.83	481.1719	217.0859/185.0509	0.05	C_23_H_29_O_11_
OLC+ H_2_O + glucuronide	5.74	497.1666	321.1344/201.0759	0.17	C_23_H_29_O_12_

**Table 2:** Permeability coefficients (*P*_eff_) and apparent permeability coefficients (*P*_app_) of oleocanthal and levofloxacin. Results are expressed as the mean ± SD of *n* = 4. Data are normalised to a 10-cm intestinal segment.

Test Compound	*P*_eff_ (× 10^−5^ cm/s)	*P*_app_ (× 10^−6^ cm/s)
OLC	2.23 ± 3.16	4.12 * ± 2.33
LEV	7.64 ± 5.55	10.91 ± 6.27

* *p* < 0.05, Mann–Whitney *U*-test.

## Supplementary Materials

The following are available online at https://www.mdpi.com/1999-4923/12/2/134/s1, Table S1: The declustering potential (DP), focusing potential (FP), collision energy (CE) and entrance potential (EP) settings for the oleocanthal and metabolites.
