# Correction: López-Yerena, A., et al. “Absorption and Intestinal Metabolic Profile of Oleocanthal in Rats” *Pharmaceutics* 2020, *12*, 134

**DOI:** 10.3390/pharmaceutics12121220

**Published:** 2020-12-17

**Authors:** Anallely López-Yerena, Anna Vallverdú-Queralt, Raf Mols, Patrick Augustijns, Rosa M. Lamuela-Raventós, Elvira Escribano-Ferrer

**Affiliations:** 1 Nutrition, Food Science and Gastronomy Department, XaRTA, Institute of Nutrition and Food Safety (INSA-UB), School of Pharmacy and Food Sciences, University of Barcelona, 08028 Barcelona, Spain; naye.yerena@gmail.com (A.L.-Y.); avallverdu@ub.edu (A.V.-Q.); lamuela@ub.edu (R.M.L.-R.); 2 CIBER Physiopathology of Obesity and Nutrition (CIBEROBN), Institute of Health Carlos III, 28029 Madrid, Spain; 3 Drug Delivery and Disposition, KU Leuven, 3000 Leuven, Belgium; raf.mols@kuleuven.be (R.M.); patrick.augustijns@pharm.kuleuven.be (P.A.); 4 Department of Pharmacy and Pharmaceutical Technology and Physical Chemistry, Biopharmaceutics and Pharmacokinetics Unit, Institute of Nanoscience and Nanotechnology (IN2UB), Pharmacy and Food Sciences School, University of Barcelona, 08028 Barcelona, Spain

The authors would like to make the following corrections to this paper [1]:

## 1. Change in Equation (1)

We have found two typographical errors in Equation (1) and we wish to replace

$$P_{\mathrm{eff}}=\frac{-{Ø}_{\mathrm{in}}}{2\pi RL}\times Ln\;\frac{C_{\mathrm{in}}}{C_{\mathrm{out}.\mathrm{cor}}}$$

With

$$P_{\mathrm{eff}}=\frac{-{Ø}_{\mathrm{in}}}{2\pi RL}\times Ln\;\frac{C_{\mathrm{out}.\mathrm{cor}}}{C_{\mathrm{in}}}$$

## 2. Change in Table 2

We have found a typographical error in the *P*_eff_ heading in Table 2. In addition, and after reviewing the calculations again, we have detected that the data shown in Table 2 for levofloxacin did not consider the normalization of the 10-cm intestinal segment. With the normalization, the *P*_eff_ value for levofloxacin increases (according to the length of the intestinal segment used). For these reasons, the authors wish to replace 

**Table 2:** Permeability coefficients (*P*_eff_) and apparent permeability coefficients (*P*_app_) of oleocanthal and levofloxacin. Results are expressed as the mean ± SD of n = 4. Data are normalised to a 10-cm intestinal segment.

Test Compound	*P*_eff_ (×10^−5^ cm/s)	*P*_app_ (×10^−6^ cm/s)
OLC	2.23 ± 3.16	4.12 * ± 2.33
LEV	7.64 ± 5.55	10.91 ± 6.27

* *p* < 0.05, Mann-Whitney U-test.

With

**Table 2:** Permeability coefficients (*P*_eff_) and apparent permeability coefficients (*P*_app_) of oleocanthal and levofloxacin. Results are expressed as the mean ± SD of *n* = 4. Data are normalised to a 10-cm intestinal segment.

Test Compound	*P*_eff_ (×10^−4^ cm/s)	*P*_app_ (×10^−6^ cm/s)
OLC	2.85 ± 2.63	4.12 * ± 2.33
LEV	12.69 ± 6.64	10.91 ± 6.27

* *p* < 0.05, Mann–Whitney U-test.

These changes do not affect the discussion and conclusions of the study. The authors would like to apologize for any inconvenience caused to the readers by these changes.
