# Supplementary material for: Absorption and Intestinal Metabolic Profile of Oleocanthal in Rats
Source: Pharmaceutics. 2020 Feb 5;12(2):134. doi: 10.3390/pharmaceutics12020134 (PMC7076358; doi:10.3390/pharmaceutics12020134)
Supplement: Supplementary file 1 [file pharmaceutics-12-00134-s001.pdf]

pharmaceutics

MDPI

Supplementary Materials: Absorption and Intestinal Metabolic Profile of Oleocanthal in Rats

Anallely López-Yerena, Anna Vallverdú-Queralt, Raf Mols, Patrick Augustijns, Rosa M. Lamuela-Raventós and Elvira Escribano-Ferrer

Table S1. The declustering potential (DP), focusing potential (FP), collision energy (CE) and entrance potential (EP) settings for the oleocanthal and metabolites.

Compound	RT (min)	MS/MS	DP (V)	FP (V)	CE (v)	EP (v)	LOQ (μg/mL)	LOD (μg/mL)
OLC	5.8	303–285/179	−40	−170	−10	−5	0.27	0.08
OLC-hydroxylated	6.4	319–153/183	−30	−170	−30	−5	0.20	0.06
OLC-hydrated	6.3	321–201/183	−40	−170	−10	−5	-	-
OLC-hydrogenated + glucuronidation	5.8	481–217/185	−40	−170	−10	−5	-	-
OLC-hydrated + glucuronidation	5.7	497–321/201	−40	−170	−10	−5	-	-

RT—retention time; LOD—limit of detection; LOQ—limit of quantification. [a] Identified with standard.
